# Subsequent shock deliveries are associated with increased favorable neurological outcomes in cardiac arrest patients who had initially non-shockable rhythms

**DOI:** 10.1186/s13054-015-1028-0

**Published:** 2015-09-10

**Authors:** Nobuya Kitamura, Taka-aki Nakada, Koichiro Shinozaki, Yoshio Tahara, Atsushi Sakurai, Naohiro Yonemoto, Ken Nagao, Arino Yaguchi, Naoto Morimura, on behalf of the SOS-KANTO 2012 Study Group

**Affiliations:** Department of Emergency and Critical Care Medicine, Kimitsu Chuo Hospital, 1010 Sakurai, Kisarazu-City, Chiba 292-8535 Japan; Department of Emergency and Critical Care Medicine, Chiba University Graduate School of Medicine, 1-8-1 Inohana, Chuo-ku, Chiba-City, Chiba 260-8677 Japan; National Cerebral and Cardiovascular Center Hospital, 5-7-1 Fujishiro-dai, Suita, Osaka 565-8565 Japan; Division of Emergency and Critical Care Medicine, Department of Acute Medicine, Nihon University School of Medicine, 30-1 Oyaguchikamicho, Itabashi-ku, Tokyo 173-0032 Japan; National Center of Neurology and Psychiatry, Translational Medical Center, 4-1-1 Ogawa-Higashi, Kodaira, Tokyo 187-8551 Japan; Nihon University Surugadai Hospital, 1-6 Kanda-Surugadai, Chiyoda-ku, Tokyo 101-8309 Japan; Department of Critical Care and Emergency Medicine, Tokyo Women’s Medical University, 8-1 Kawadacho, Shinjuku-ku, Tokyo 162-8666 Japan; Department of Emergency Medicine, Yokohama City University Medical Center, 4 -57 Urafunecho, Minami-ku, Yokohama-City, Kanagawa 232-0024 Japan

## Abstract

**Introduction:**

Previous studies evaluating whether subsequent conversion to shockable rhythms in patients who had initially non-shockable rhythms was associated with altered clinical outcome reported inconsistent results. Therefore, we hypothesized that subsequent shock delivery by emergency medical service (EMS) providers altered clinical outcomes in patients with initially non-shockable rhythms.

**Methods:**

We tested for an association between subsequent shock delivery in EMS resuscitation and clinical outcomes in patients with initially non-shockable rhythms (n = 11,481) through a survey of patients after out-of-hospital cardiac arrest in the Kanto region (SOS-KANTO) 2012 study cohort, Japan. The primary investigated outcome was 1-month survival with favorable neurological functions. The secondary outcome variable was the presence of subsequent shock delivery. We further evaluated the association of interval from initiation of cardiopulmonary resuscitation to shock with clinical outcomes.

**Results:**

In the univariate analysis of initially non-shockable rhythms, patients who received subsequent shock delivery had significantly increased frequency of return of spontaneous circulation, 24-hour survival, 1-month survival, and favorable neurological outcomes compared to the subsequent not shocked group (*P* <0.0001). In the multivariate logistic regression analysis, subsequent shock was significantly associated with favorable neurological outcomes (vs. not shocked; adjusted *P* = 0.0020, odds ratio, 2.78; 95 % confidence interval, 1.45–5.30). Younger age, witnessed arrest, initial pulseless electrical activity rhythms, and cardiac etiology were significantly associated with the presence of subsequent shock in patients with initially non-shockable rhythms.

**Conclusions:**

In this study of cardiac arrest patients with initially non-shockable rhythms, patients who received early defibrillation by EMS providers had increased 1-month favorable neurological outcomes.

**Electronic supplementary material:**

The online version of this article (doi:10.1186/s13054-015-1028-0) contains supplementary material, which is available to authorized users.

## Introduction

Shockable rhythms, including ventricular fibrillation (VF) and pulseless ventricular tachycardia (VT) on the initial electrocardiography (ECG), have long been recognized as key factors in promoting favorable neurological outcomes in cardiac arrest (CA) [1–3]. Substantial studies have shown risk factors associated with clinical outcomes of patients who have initially shockable rhythms [1, 4], while less attention has been paid to initially nonshockable rhythms, such as pulseless electrical activity (PEA) and asystole [5]. However, the number of patients with initially nonshockable rhythms is greater than those with shockable rhythms [6, 7]. Furthermore, patients with initial nonshockable rhythms have poorer prognoses [8, 9]. Thus, it is of great importance to study CA patients with initially nonshockable rhythms in detail to improve the fatality rate of CA patients [5, 10].

Initially nonshockable rhythms in CA patients can be converted to shockable rhythms through cardiopulmonary resuscitation (CPR) [11, 12]. It is believed that treatment for nonshockable rhythms should focus on increasing cardiac muscle perfusion and myocardial tissue excitability with CPR to attain a subsequent conversion to shockable rhythms, some of which can be treated effectively by defibrillation [13]. However, Hallstrom et al. [11] reported an association between subsequent shock delivery by emergency medical service (EMS) providers and decreased hospital survival, which has led to controversy. Subsequently, three studies [14–16] on this topic showed results in contradiction to the report from Hallstrom et al. [11].

More recently, Thomas et al. [12] studied risk factors of survival in patients with initially nonshockable rhythms and reported no significant association between subsequent EMS shock deliveries and increased hospital survival, while Goto et al. [17], in contrast, reported that subsequent shock delivery was significantly associated with increased 1-month favorable neurological outcome in patients with initially nonshockable rhythms. Despite the findings of these six studies on initially nonshockable rhythms [11, 12, 14–17], whether shock delivery during EMS resuscitation is associated with altered clinical outcomes in CA patients is still unclear. In addition, few reports have studied the etiology of CA and intervals between CPR and first shock delivery by EMS providers in patients with initially nonshockable rhythms in detail.

Therefore, we first tested for an association between subsequent shock delivery during EMS resuscitation and altered 1-month neurological outcomes in patients with initially nonshockable rhythms as a primary analysis. We further investigated factors associated with the presence of subsequent shock delivery, particularly regarding the etiology of CA, using multivariate regression analysis. We also evaluated the association of the interval between initiation of CPR and EMS shock with clinical outcomes. This study used a large, multicenter cohort collected for the Survey of Survivors after Out-of-hospital Cardiac Arrest in the Kanto Region (SOS-KANTO) 2012 Study Group; data from this cohort were prospectively collected by EMS personnel and hospital staff.

## Materials and methods

### Study design

The SOS-KANTO 2012 study was prospectively conducted to accumulate prehospital and inhospital records for CA patients in the Kanto region, including Tokyo Prefecture, Japan, with the support of the Kanto Regional Group of the Japanese Association for Acute Medicine [18]. The SOS-KANTO 2012 study included 16,452 CA patients from 67 emergency medical centers between January 2012 and March 2013. The relevant institutional review boards of all institutions approved the study (see Additional file 1 for details). The review boards waived the need for written informed consent.

### Patients

**Fig. 1:**
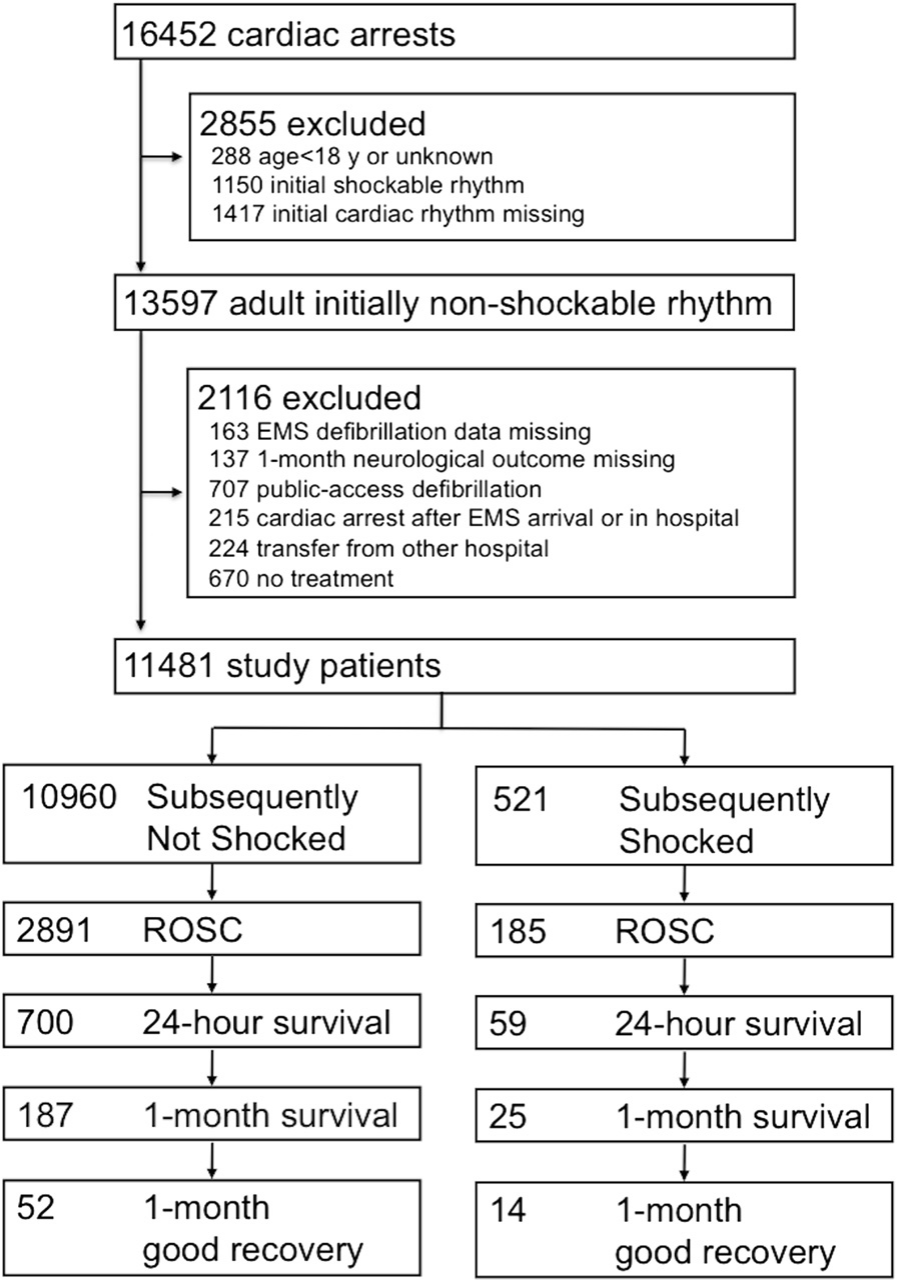
Flow diagram of the study population. A total of 16,452 CA patients were enrolled in the SOS-KANTO 2012 study. Of these, 13,597 adult patients had initially nonshockable rhythms. Of these, 11,481 patients were evaluated in this study. Of these, 10,960 patients received no shock (Subsequently Not Shocked group) and 521 patients received shock(s) during EMS resuscitation (Subsequently Shocked group). *1-month good recovery* survival with favorable neurological outcome defined as Cerebral Performance Category of 1 or 2 at 1 month after CA, *EMS* emergency medical service, *ROSC* return of spontaneous circulation

The current study included adult CA patients (≥18 years of age) who fit the following criteria: presented with an initial EMS-monitored nonshockable rhythm (PEA or asystole), received CPR administered by EMS providers, and were subsequently transported to one of the participating institutions. Exclusion criteria were as follows: absence of data regarding inclusion criteria or main outcomes (i.e., initially EMS-monitored ECG, EMS defibrillation data, and 1-month neurological outcomes); receipt of public-access defibrillation; onset of CA subsequent to the arrival of paramedics or at the hospital; transfer from another hospital; and no treatment performed at the participant hospital without the achievement of return of spontaneous circulation (ROSC). A total of 16,452 CA patients were enrolled in the SOS-KANTO 2012 study (Fig. 1). Of these, 13,597 adult patients had initially nonshockable rhythms. Of these, 2116 patients met the exclusion criteria, and thus 11,481 patients were evaluated in this study (Fig. 1).

### Data collection and definition

EMS providers collected prehospital information in the standardized Utstein style, including age, sex, location, witnessed arrest, bystander CPR, call–response interval, initial cardiac rhythms monitored by EMS providers, and shock deliveries by EMS providers. We defined rhythm conversion of nonshockable rhythms to VF/ pulseless VT during EMS resuscitation when EMS providers delivered shock(s) for patients who had initially nonshockable rhythms; we used shock delivery as a surrogate indicator of conversion [11, 12, 14–17]. The call–response interval was defined as the interval from receipt of a call by emergency response dispatchers to the time when the emergency response vehicle came to a stop. Shock delivery time was defined as the interval between the initiation of CPR by EMS providers and the time of first shock delivery by EMS providers.

Patients who had initially nonshockable rhythms and subsequently received shock(s) during EMS resuscitation were assigned to the “Subsequently Shocked” group, while other patients who did not receive subsequent shock by EMS providers were assigned to the “Subsequently Not Shocked” group. EMS providers used semiautomated external defibrillators to analyze the rhythm, and if a shock was indicated it was delivered [19].

Physicians were responsible for treating the patient-determined causes of CA, including cardiac and noncardiac (asphyxia, trauma, aortic disease, drawing, cerebrovascular disease, drug overdose, and others). The institutional researchers collected information that included ROSC during resuscitation by EMS providers or physicians, 24-hour survival, 1-month survival, and neurological outcomes. Neurological outcomes were evaluated using the Cerebral Performance Categories (CPCs) [20]; responses were scored as follows: CPC 1, good cerebral performance; CPC 2, moderate cerebral disability; CPC 3, severe cerebral disability; CPC 4, coma/vegetative state; and CPC 5, death. Favorable neurological outcome was defined as CPC 1 or 2.

### Statistical analysis

The primary outcome variable was 1-month survival with a favorable neurological outcome. For the primary analysis, we assessed differences in 1-month favorable neurological outcomes by subsequent shock delivery using a multivariate logistic regression to allow for adjustment for potential confounding factors reported previously, including age, sex, public location, witnessed arrest, bystander CPR, call–response interval, initial PEA rhythm, and cardiac etiology as covariates [11, 12, 14–17]. For the secondary analysis, we used a multivariate logistic regression to assess factors associated with the presence of subsequent shock. In addition, the frequency of ROSC, 24-hour survival, 1-month survival, and 1-month favorable neurological outcome in subsequent shock patients were compared by interval of EMS shock delivery. Odds ratios (ORs) and 95 % confidence intervals (CIs) were calculated. The level of significance was set at α = 0.05 with a two-tailed test. Analyses were performed using SPSS (version 21; SPSS, Chicago, IL, USA) statistical software packages.

## Results

Of 11,481 patients who had initially nonshockable arrest rhythms monitored by EMS providers, 521 patients received shock(s) during EMS resuscitation (Subsequently Shocked group) and 10,960 patients received no shock (Subsequently Not Shocked group) (Table 1). Patients who received subsequent shocks were younger than patients who were not shocked. The frequencies of male sex, witnessed arrest, initial PEA rhythms, and cardiac etiology in patients who were shocked were higher compared with those who were not shocked (Table 1).

**Table 1:** Baseline characteristic of patients who had initially nonshockable arrest rhythms

	Subsequently	Subsequently	
	Not Shocked^a^	Shocked^b^	
	(*n* = 10,960)	(*n* = 521)	*P* value
Age (years)	71.2 (16.9)	68.0 (16.5)	<0.0001
Sex (% male)	58.5	65.3	0.0024
Public location (%)	18.8	20.9	0.24
Witnessed arrest (%)	42.2	54.5	<0.0001
Bystander CPR (%)	30.7	28.1	0.21
Call–response interval (minutes)	8.0 (3.6)	8.2 (3.8)	0.18
Initial rhythm PEA (*n* (%))	2455 (22.4)	229 (44.0)	<0.0001
Initial rhythm asystole (*n* (%))	8505 (77.6)	292 (56.0)	<0.0001
Shock delivery time (minutes)	–	13.0 (9.8)	N/A
Etiology (*n* (%))			
Cardiac	4748 (43.3)	331 (63.5)	<0.0001
Noncardiac	6212 (56.7)	190 (36.5)	
Asphyxia	1469(13.4)	39 (7.5)	
Trauma	928 (8.5)	11 (2.1)	
Aortic disease	569 (5.2)	24 (4.6)	
Drowning	447 (4.1)	12 (2.3)	
Cerebrovascular disease	267 (2.4)	11 (2.1)	
Drug overdose	72 (0.7)	3 (0.6)	
Others or unknown	2460 (22.2)	90 (17.3)	

Data are mean (standard deviation) for continuous variables. *P* values calculated using the *t* test and the chi-square test

^a^Patients who had initially nonshockable rhythms and received no shock(s) during EMS resuscitation

^b^Patients who had initially nonshockable arrest rhythms and subsequently received shock(s) owing to conversion to shockable rhythms during EMS resuscitation

*CPR* cardiopulmonary resuscitation, *EMS* emergency medical service, *N/A* not available, *PEA* pulseless electrical activity

In the univariate analysis, patients in the Subsequent Shock group had significantly increased frequency of ROSC, 24-hour survival, 1-month survival, and favorable neurological outcomes compared with the Subsequent Not Shocked group (*P* <0.0001) (Table 2). In the primary analysis of this study population with initially nonshockable rhythms, patients who had subsequent shocks by EMS providers had significantly increased 1-month favorable neurological outcomes compared with those who received no subsequent shock in a multivariate logistic regression analysis adjusting for potential confounding factors, including age, sex, public location, witnessed arrest, bystander CPR, call–response interval, initial PEA rhythm, and cardiac etiology (adjusted *P* = 0.0020; OR, 2.78; 95 % CI, 1.45–5.30) (Table 3).

**Table 2:** Clinical outcomes between the Subsequently Shocked and Not Shocked groups

	Subsequently	Subsequently		
	Not Shocked^a^	Shocked^b^	Odds ratio	
	(*n* = 10,960)	(*n* = 521)	(95 % CI)	*P* value
ROSC	2891 (26.4)	185 (35.5)	1.54 (1.28–1.86)	<0.0001
24-hour survival	700 (6.4)	59 (11.3)	1.87 (1.41–2.48)	<0.0001
1-month survival	187 (1.7)	25 (4.8)	2.90 (1.89–4.45)	<0.0001
1-month good recovery^c^	52 (0.5)	14 (2.7)	5.79 (3.19–10.5)	<0.0001

Data presented as number (percentage). *P* values calculated using chi-square test

^a^Patients who had initially nonshockable rhythms and received no shock(s) during EMS resuscitation

^b^Patients who had initially nonshockable arrest rhythms and subsequently received shock(s) owing to conversion to shockable rhythms during EMS resuscitation

^c^Survival with favorable neurological outcome defined as Cerebral Performance Category of 1 or 2 at 1 month after cardiac arrest

*CI* confidence interval, *EMS* emergency medical service, *ROSC* return of spontaneous circulation

**Table 3:** Multivariate analysis for factors associated with favorable neurological outcome at 1 month after cardiac arrest in patients with initially nonshockable rhythms

	Odds ratio (95 % CI)	*P* value
Age (per year)	0.97 (0.96–0.98)	<0.0001
Male	0.99 (0.58–1.69)	0.97
Public location	1.54 (0.90–2.62)	0.11
Witnessed arrest	1.30 (0.75–2.24)	0.35
Bystander CPR	0.83 (0.47–1.48)	0.54
Call–response interval^a^ (per minute)	0.91 (0.83–0.99)	0.037
Initial rhythm PEA	11.3 (5.94–21.6)	<0.0001
Cardiac etiology	1.82 (1.07–3.09)	0.028
Subsequently shocked	2.78 (1.45–5.30)	0.0020

*P* values calculated using a multivariate logistic regression

^a^Shock delivery time was the interval from the initiation of CPR by EMS providers to the first shock delivery by EMS providers

*CI* confidence interval, *CPR* cardiopulmonary resuscitation, *EMS* emergency medical service, *PEA* pulseless electrical activity

**Fig. 2:**
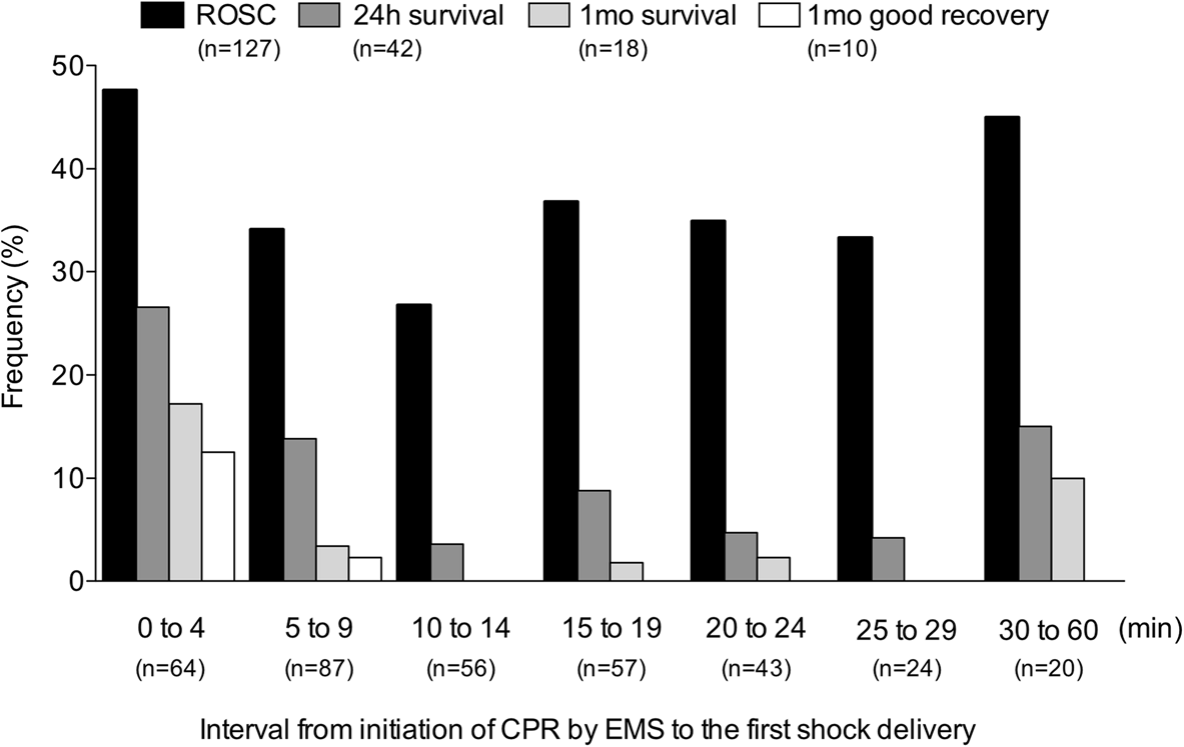
Clinical outcomes by interval between CPR and initial shock in subsequent shock patients. There were significant decreases in 24-hour survival, 1-month survival, and 1-month favorable neurological outcome according to the interval between initiation of cardiopulmonary resuscitation (*CPR*) and initial shock delivery by emergency medical service providers (*EMS*) in patients who received subsequent shock (ROSC, *P* = 0.58; 24-hour survival, *P* = 0.0032; 1-month survival, *P* = 0.013; 1-month good recovery, *P* = 0.0002). *1-month good recovery* survival with favorable neurological outcome defined as CPC of 1 or 2 at 1 month after CA. *P* values calculated using the chi-square test for trend. *1mo* 1-month, *24h* 24-hour, *ROSC* return of spontaneous circulation

We next examined factors associated with the presence of subsequent shock. Younger age, witnessed arrest, having initial PEA rhythms, and cardiac origin of etiology were significantly associated with increased subsequent shock (Table 4). In the Subsequently Shock group, the frequencies of patients with 24-hour survival, 1 month survival, and 1-month favorable neurological outcome significantly deceased over time, which is the interval from initiation of CPR to shock delivery (test for trend; 24-hour survival, *P* = 00032; 1-month survival, *P* = 0.013; 1-month favorable neurological outcome, *P* = 0.0002); while there was no difference in the frequencies of patients with ROSC over time (test for trend; *P* = 0.58) (Fig. 2). Patients with 1-month favorable neurological outcomes received subsequent shock deliveries within 9 minutes of initiation of CPR (Fig. 2).

**Table 4:** Multivariate analysis for factors associated with subsequent shock in emergency medical service resuscitation in patients with initial nonshockable rhythms

	Odds ratio (95 % CI)	*P* value
Age	0.98 (0.97–0.98)	<0.0001
Male	0.85 (0.70–1.03)	0.11
Public location	1.12 (0.89–1.41)	0.34
Witnessed arrest	1.37 (1.12–1.66)	0.0018
Bystander CPR	0.90 (0.73–1.10)	0.30
Call–response interval	1.02 (1.00–1.04)	0.13
Initial rhythm PEA	2.67 (2.19–3.25)	<0.0001
Etiology		
Cardiac (reference)	1	
Asphyxia	0.30 (0.21–0.43)	<0.0001
Trauma	0.09 (0.05–0.18)	<0.0001
Aortic disease	0.46 (0.29–0.71)	0.00047
Drowning	0.53 (0.29–0.95)	0.0330
Cerebrovascular disease	0.40 (0.22–0.75)	0.0044
Drug overdose	0.44 (0.14–1.42)	0.17
Others or unknown	0.47 (0.37–0.60)	<0.0001

*P* values calculated using a multivariate logistic regression

*CI* confidence interval, *CPR* cardiopulmonary resuscitation, *PEA* pulseless electrical activity

## Discussion

This study of initially nonshockable rhythms demonstrated that patients who received subsequent shock had increased 1-month favorable neurological outcomes compared with those who received no shock from EMS providers.

The association of subsequent shock deliveries with altered clinical outcomes in patients of initially nonshockable rhythms has been analyzed in six studies (Table 5) [11, 12, 14–17]. Of four studies published prior to 2009, three studies reported an association of subsequent shock deliveries with favorable clinical outcomes [14–16], and one study reported the opposite result [11]. More recently, Thomas et al. [12] reported no significant association of subsequent shock delivery with survival to hospital discharge in patients with initially nonshockable rhythms (OR, 0.88; 95 % CI, 0.60–1.30). Goto et al. [17] published findings that patients with subsequent shock deliveries had increased 1-month favorable neurological outcomes. Thus, of seven studies including the present work, five studies showed that subsequent shock delivery was associated with increased favorable clinical outcomes (Table 5).

**Table 5:** Studies of subsequent shock in patients with initially nonshockable rhythms

	Hallstrom et al. [11]	Herlitz et al. [14]	Kajino et al. [15]	Olasveemgen et al. [16]	Thomas et al. [12]	Goto et al. [17]	SOS-KANTO Study Group [18]
Published year	2007	2008	2008	2009	2013	2014	2015
Sample size (*n*)	738	22,465	12,353	753	6556	569,937	11,481
Response time (minutes)	6.0 ± 2.6	7 ^a^	6.0 ± 2.3	7 (3–11) ^a^	–	7 (5–9)^a^	8.2 ± 3.8
Shock delivery time^b^ (minutes)	21.0 ± 8.1	–	12.3 ± 6.9	–	–	20 (15–27)^a^	13.0 ± 9.8
Country	USA	Sweden	Japan	Norway	USA	Japan	Japan
Subsequent shock (%)	22.2	26.0	3.9	13.0	18.9	4.8	4.5
Association of subsequent shock with outcomes^c^	Unfavorable outcomes	Favorable outcomes	Favorable outcomes	Favorable outcomes	No difference	Favorable outcomes	Favorable outcomes

^a^Data are median (interquartile range) for continuous variables

^b^Shock delivery time was the interval from the initiation of CPR by EMS providers to the first shock delivery by EMS providers

^c^Association of subsequent shock with increased unfavorable or favorable clinical outcome

*CPR*, cardiopulmonary resuscitation, *EMS* emergency medical service, *SOS-KANTO*, survey of survivors after out-of-hospital cardiac arrest in the Kanto region

We assessed the association between the interval from the initiation of CPR by EMS providers to the first shock delivery and clinical outcomes. As shown in Fig. 2, frequencies of ROSC remained over 30 % after a 20-minute interval from the initiation of CPR by EMS providers to the first shock delivery in the subsequent shock group. However, frequencies of 1-month favorable neurological outcomes decreased over time. A previous study showed that receiving subsequent defibrillation earlier (<20 minutes) rather than later (≥20 minutes) was associated with increased favorable outcomes for CA patients [17]. Similarly, in the current study, patients with 1-month favorable neurological outcomes had received subsequent shock deliveries earlier rather than later (Fig. 2).

As shown in Table 5, there are regional differences in the prevalence of subsequent shock deliveries in patients with initially nonshockable rhythms. The frequency of subsequent shock in Japan was lower than that in Europe, the United States (Table 5), and Australia [21]. EMS providers in Japan have an obligation to transfer out-of-hospital CA patients, except patients with decapitation, transection of the trunk, or postmortem changes such as rigor mortis, postmortem lividity, and cloudiness of the cornea [22]. Different EMS systems may thus result in different frequencies of subsequent conversion from nonshockable to shockable rhythms across countries. Increased favorable outcomes in patients with subsequent shock were reported in Japan and Europe, while the opposite results were obtained from the United States, suggesting regional differences may be a considerable factor in understanding the association between subsequent shock and altered clinical outcomes.

We found that either younger age or initial PEA rhythms were associated with increased favorable neurological outcomes in patients with initially nonshockable rhythms (Table 4). The association between younger age or initial PEA rhythms and increased favorable outcomes was observed in all six studies of initially nonshockable rhythms from different regions [11, 12, 14–17]. Age and initial PEA rhythms are therefore likely to be important outcome predictors across countries.

Thomas et al. [12] hypothesized that the inconsistent association between subsequent shock and clinical outcome may be caused by different etiologies of CA. In a subsequent study, Goto et al. [17] tested for an association of altered clinical outcomes with subsequent shock including presumed cardiac cause or not as a covariate, which was collected by EMS personnel; they reported an association between presumed cardiac cause and increased 1-month favorable outcomes (OR, 1.28; 95 % CI, 1.20–1.37). In their discussion, they also pointed out the insufficiency of relevant information to analyze etiologies more precisely owing to their prehospital-based dataset [17]. Etiologies in the current study were precisely collected by physicians; cardiac etiology was significantly associated with increased 1-month favorable neurological outcome in initially nonshockable rhythms (*P* = 0.028; OR, 1.82; 95 % CI, 1.07–3.09) (Table 3). Moreover, the cardiac etiology was significantly associated with increased prevalence of subsequent shock (Table 4), suggesting that cardiac etiology is likely to be of importance in patients with initially nonshockable rhythms.

This study has some limitations. First, the integrity and validity of the data, unmeasured confounders, as well as ascertainment bias are potential limitations of our observational study. The use of uniform Utstein template data collection, which was developed by international experts, and the large sample size should minimize these potential sources of bias. Second, this study was hospital driven but was not a population-based study; therefore, our findings might not be representative of all hospitals or other local regions. However, the large number of participating hospitals should minimize this limitation. Fourth, as in previous studies [11, 12, 14–17], we tested for an association of altered clinical outcome with EMS shock delivery, which is a surrogate indicator of rhythm conversion of nonshockable rhythms to VF/ pulseless VT. In real-world emergency situations, there are inevitably some violations of protocol or just mistaken rhythm identifications. Even though the EMS providers used semiautomated external defibrillators during the EMS resuscitation, there was a very small possibility that inappropriate shocks were given. The significant association of altered clinical outcome with EMS shock in this study is thus not likely to prove the association of rhythm conversions with altered outcome.

## Conclusions

In this study of CA patients with initially nonshockable rhythms, patients who received early defibrillation by EMS had increased 1-month favorable neurological outcomes. In patients with initially nonshockable rhythms, cardiac etiology, younger age, witnessed arrest, and initial PEA rhythms were associated with increased subsequent shock.

## Key messages

- In this study of cases of initially nonshockable CA rhythms, patients who received subsequent shock by EMS providers had significantly increased 1-month favorable neurological outcomes compared with those who received no subsequent shock.
- Cardiac etiology was associated with the presence of subsequent shock and increased 1-month favorable neurological outcome in patients with initially nonshockable rhythms.

## Additional file

Additional file 1:
**Presents a list of institutional review boards.** (PDF 77 kb)

## Abbreviations

CA: Cardiac arrest
CI: Confidence interval
CPC: Cerebral Performance Category
CPR: Cardiopulmonary resuscitation
ECG: Electrocardiography
EMS: Emergency medical service
OR: Odds ratio
PEA: Pulseless electrical activity
ROSC: Return of spontaneous circulation
SOS-KANTO: Survey of Survivors after Out-of-hospital Cardiac Arrest in the Kanto Region
VF: Ventricular fibrillation
VT: Ventricular tachycardia
